# Space-time modelling of monthly malaria incidence for seasonal associated drivers and early epidemic detection in Southern Ethiopia

**DOI:** 10.1186/s12936-023-04742-9

**Published:** 2023-10-09

**Authors:** Yonas Shuke Kitawa, Zeytu Gashaw Asfaw

**Affiliations:** 1 Department of Statistics, College of Natural and Computational Sciences, Hawassa University, Hawassa, Ethiopia; 2 Department of Bio-statistics and Epidemiology, School of Public Health, Addis Ababa University, Addis Ababa, Ethiopia

**Keywords:** Malaria Epidemic Early Warning (MEWS), Spatial time series, Malaria, Disease mapping, Monte Carlo maximum likelihood, *P. falciparum*

## Abstract

**Background:**

Although Ethiopia has made great strides in recent years to reduce the threat of malaria, the disease remains a significant issue in most districts of the country. It constantly disappears in parts of the areas before reappearing in others with erratic transmission rates. Thus, developing a malaria epidemic early warning system is important to support the prevention and control of the incidence.

**Methods:**

Space-time malaria risk mapping is essential to monitor and evaluate priority zones, refocus intervention, and enable planning for future health targets. From August 2013 to May 2019, the researcher considered an aggregated count of genus *Plasmodium falciparum *from 149 districts in Southern Ethiopia. Afterwards, a malaria epidemic early warning system was developed using model-based geostatistics, which helped to chart the disease’s spread and future management.

**Results:**

Risk factors like precipitation, temperature, humidity, and nighttime light are significantly associated with malaria with different rates across the districts. Districts in the southwest, including Selamago, Bero, and Hamer, had higher rates of malaria risk, whereas in the south and centre like Arbaminch and Hawassa had moderate rates. The distribution is inconsistent and varies across time and space with the seasons.

**Conclusion:**

Despite the importance of spatial correlation in disease risk mapping, it may occasionally be a good idea to generate epidemic early warning independently in each district to get a quick picture of disease risk. A system like this is essential for spotting numerous inconsistencies in lower administrative levels early enough to take corrective action before outbreaks arise.

## Introduction

The risk of malaria has considerably decreased during the past few years in various parts of the world. Notwithstanding the recent signal of re-emergency [1], several regions with moderate to high prevalence are successful in reducing the burden of malaria. The development of drug-resistant parasites, the urgency of other pandemics, and the deterioration of programmes designed for malaria control, as stated by [2], have all been factors in the recent comeback of malaria. The COVID-19 pandemic re-emergency had a major impact on the world’s population in general and Africa in particular. There is an urgent need to speed up national eradication efforts to meet the 2030 countries’ elimination target [3] by concentrating on high-burden areas and considering places with a signal of an outbreak.

Since 1958 in Ethiopia, several serious malaria epidemics have occurred for approximately 5 to 8 years in most lowland and some highland areas up to 2,500 ms of elevation [4]. Meteorological, environmental, and socioeconomic factors like; rainfall, temperature, humidity, and others are associated with such risk [5, 6]. There is evidence of malaria resurgence from district to district and over time, as described in various literature, including [5, 7, 8] and [9]. Around 60 % of the population and 75 % of the country’s territory are at risk of malaria, with *Plasmodium falciparum* accounting for roughly 65–75% of all cases that have been documented [1, 10]. With an unstable seasonal transmission of incidence occurring from September to mid-December, immediately after the main rainy season, and a minor transmission season occurring between March and May [11, 12], the country is thought to have low to moderate malaria transmission intensity. Within a specific geographic area, the transmission is seasonal and changes over space and time, according to [13]. This may be related to climate changes that are favorable to parasite development that significantly impacted malaria transmission. Disease risk mapping is therefore useful to identify districts with increased risks [14], as a population of all age groups is at risk with an estimated prevalence of 1.3%.

The COVID-19 [1] emergency and other health care needs which are vying for scarce resources and various political instability in Ethiopia, leads to the re-establishment of the system, regardless of whether the illness risk is declining. To enhance public health decision-making for the monitoring and prevention of malaria epidemics, it is important to build an effective malaria epidemic early warning system. By prioritizing prone locations and times that are most at risk, such a system helps with public health decisions [15]. On the other hand, using those approaches to cluster districts also supports determining the seasonality of the risk rather than using commonly specified patterns throughout the country [16, 17].

To predict disease risks and identify regions that demonstrate atypical outbreaks, many researchers have established malaria epidemic early wake-up calls [16, 18, 19]. Such tools are designed to identify at-risk districts so that preventative measures can be taken before outbreaks begin [18]. Many techniques use present or projected climatic conditions to forecast the risk of malaria in the upcoming weeks and months [16]. Due to the complexity of forecasting with an areal model, using only those covariates does not determine a clear picture of the seasonality of malaria in the area as the seasonality is often noticed irrespective of the climatic conditions of the area.

In combination with the variability of climatic conditions across the districts in the country, developing a malaria epidemic early warning system in lower administrative levels independently by including seasonality parameters helps to understand the heterogeneity of malaria in the area. Such approaches help to see the variability and understand seasonality across each district and then cluster districts based on temporal trends. Also, the approach gives stockholders in each district to update their plan taking into account district-level heterogeneity in addition to what is happening in nearby areas. While developing an early warning system, [16] takes into account log-transformed malaria cases as responses and fits ARIMA and SARMA models. Yet, transforming aggregated count into a continuous measurement has its drawbacks [20]. However, the drawback of modelling aggregated count as a continuous measurement by transformation was first noted in [20]. This is because generalized linear models are one of the better modelling alternatives for such data sets. However, when dealing with zero counts, which are common in spatial data, a log transformation of counts has additional drawbacks [20].

It is a good idea to begin clustering the disease risk at each administrative district based on the temporal trend and estimating the seasonality therein to gain initial insight into the current picture of disease risk. These models are crucial for locating and predicting risky areas so that timely preventive action may be taken, which is sometimes challenging to achieve using areal models. On the other hand, the spatial correlation is weaker to describe the relationship between surrounding districts when the distance between them is quite great. Decision-makers must therefore be capable of comprehending the complex dynamics not just in space but also in time utilizing an administrative-level epidemic early warning model to forecast threats in the next weeks or months.

One can incorporate covariates to account for seasonality when developing such models, however, others consider seasonality components on a yearly or monthly basis in addition to covariates. Yet, there is no sufficient evidence to suggest which possible alternative best helps to identify the seasonality of malaria in the area which significantly varies with space and time. Using the total number of malaria cases in Southern Ethiopia, this study aims to develop an epidemic early warning system for malaria in each administrative district. The development of such a system could involve modelling temporal correlation as a Matérn process [21, 22]. This method, which may be built by modelling malaria count as a Poisson linear mixed model, is crucial to detecting the slow underlying process and re-emergency of the risk in the area. Its objectives include:

- Identifying environmental and climatic factors associated with malaria risk in each district
- Clustering districts based on the temporal trend
- Develop a malaria epidemic early warning system that helps to detect districts with unusual risks and trends over time.

## Methods

### Study setting

**Fig. 1:**
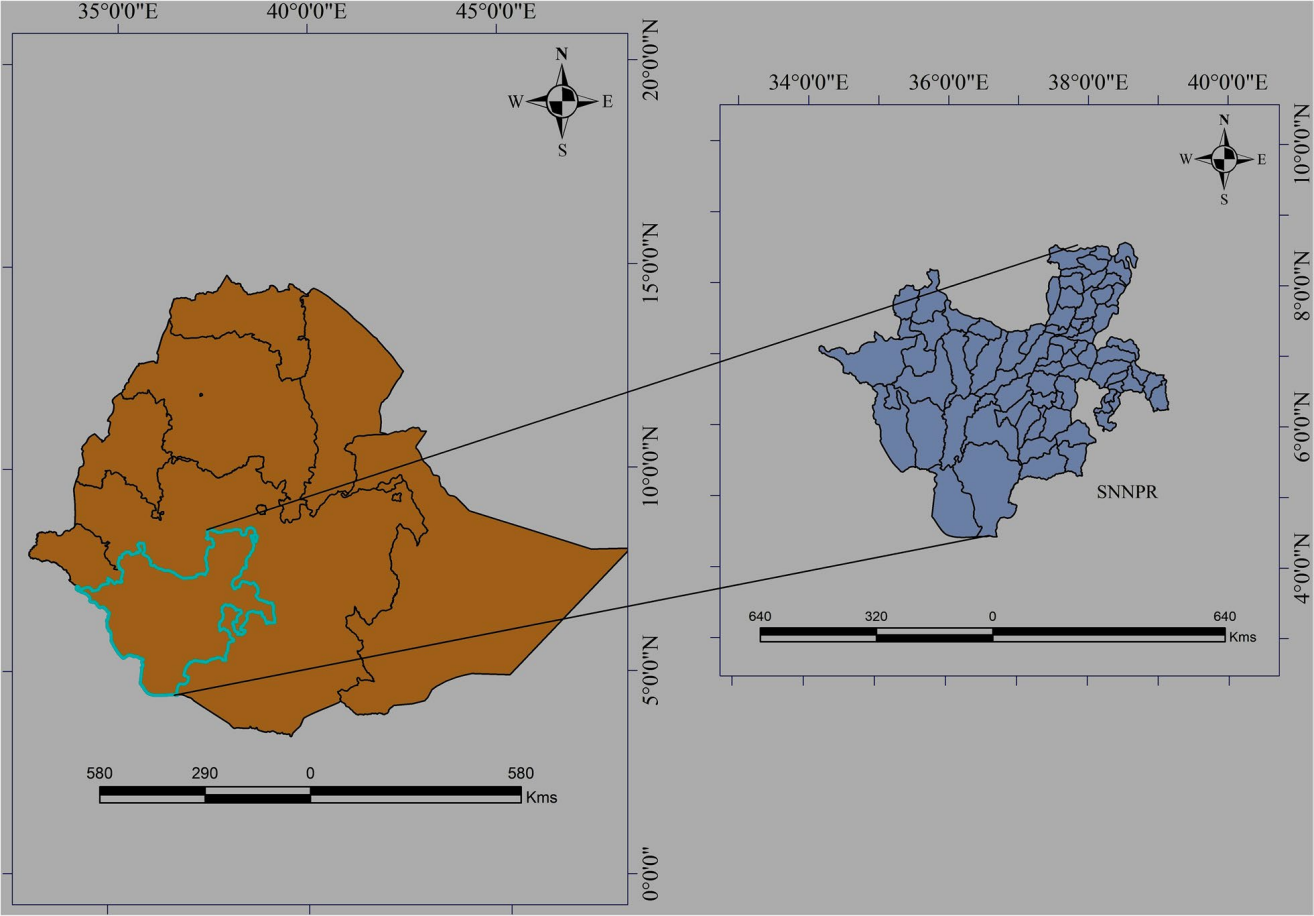
Study area map showing districts in the Southern nation nationalities and people regional state (SNNPRS), Ethiopia in 2013

The study was carried out in Southern Nation Nationalities and People Regional State (SNNPRS), in Ethiopia Fig. 1. The region is located between 6$^{\circ }$ 03’ 31.03” North latitude and 36$^{\circ }$ 43’ 38.28” East longitude. As a result of the region’s proximity to the equator, temperatures can vary from 10 degrees Celsius in high-altitude areas (4207 ms above sea level) to 28 degrees Celsius in the lowlands (360 ms above sea level). The region also has a high mean annual rainfall of 400–2200 mm. The SNNPRS is a vast land with unblemished topographical features, including the Great Rift Valley, mountains, forests, and plains. Since 2012, 149 rural districts have reported weekly malaria surveillance to the SNNPRS Public Health Institute.

### Data

The data set was obtained from the Ethiopian Public Health Institute. It consists of reported malaria counts of genus *P. falciparum* from August 2013 to May 2019 for the districts found in Southern Ethiopia. The district-level population data set was taken from the demographic department of the SNNPRS finance office and is projected based on 2007 Ethiopian census data [23]. Monthly temperature $(^{0}C)$ and total precipitation (mm) is extracted from weather and climate data provided at 2.5 min or $(\sim 21 km^{2})$ spatial resolution (worldclim.org/data/monthlywth.html and worldclim21.html). The average monthly relative humidity is derived from ECMWF Medium-Range Weather Forecasts from ERA-Interim global atmospheric reanalysis. Finally, nighttime light (NTL) is obtained from NOAAs (National Centers for Environmental Information), Visible Infrared Imaging Radiometer Suite (https://ngdc.noaa.gov/eog/viirs/index.html) available at approximately 100 m at the equator.

### Statistical model

Disease risk can occasionally change across time or space, or perhaps both. When data sets are collected over a large geographic area or an extended period, it is sometimes possible to predict that the characteristics of the process $S_{it}$ could vary between districts [24]. The model can then be fitted individually for each district to assess the distribution of the incidence [16]. Suppose $y_{it}$ denotes the monthly aggregated malaria counts of genus *P. falciparum* from $i^{th}$ districts $i=1,...,149$ at time $t=1,...,70$ in months. Conditional on $S_{it}$, the aggregated count $y_{it}$ in each administrative district are mutually independent Poisson random variables with expectation $m_{it}\lambda _{it}$ given as:

1 $$\begin{aligned} \log \{\lambda _{it}\} = d_{it}^\top \beta + S_{it} \end{aligned}$$

Where $$d_{it}$$ is a vector of space-time referenced explanatory variables with associated regression coefficients $$\beta$$, $$\lambda _{it}$$$$-$$ is a malaria incidence rate and $$m_{it}$$ is an offset representing the population at risk at each administrative district. Assuming $$S_{it}$$, temporal continuous Gaussian process in $$i^{th}$$ districts, Eq. 1 can be re-expressed as:

2 $$\begin{aligned} log(\lambda _{it})=\,& {} \alpha _{0}+\alpha _{1}cos \left( \frac{2\pi it}{12} \right) +\alpha _{2}sin \left( \frac{2\pi it}{12} \right) \nonumber \\{} & {} +\alpha _{3}cos \left( \frac{2\pi it}{4} \right) +\alpha _{4}sin \left( \frac{2\pi it}{4} \right) +S_{it} \end{aligned}$$

Sometimes, it is also possible to account for seasonality through the covariates only or a combination of both. Thus, by considering the covariates also, Eq. 2 can be expressed as:

3 $$\begin{aligned} log(\lambda _{it})=\, {} \alpha _{0}+\alpha _{1}cos \left( \frac{2\pi it}{12} \right) +\alpha _{2}sin \left( \frac{2\pi it}{12} \right) \nonumber \\{} {} +\alpha _{3}cos \left( \frac{2\pi it}{4} \right) +\alpha _{4}sin \left( \frac{2\pi it}{4} \right) +d^{T}_{it}\beta +S_{it} \end{aligned}$$

Finally, the model in Eq. 2 and Eq. 3 were fitted using aggregated count data at each $i^{th}$ district in Southern Ethiopia and then compared the prediction performance. where the temporal random effect; $S_{it}$ assumed to follow stationary and isotropic Gaussian process with variance $\sigma ^{2}$ and correlation between successive time assumed to be exponential with scale parameter $\phi$ and shape parameter $\kappa$ given as:

$$\begin{aligned} corr(S_{t},S_{t}^{'})=\rho (t,t^{'},\theta ) \end{aligned}$$

Where $\theta =(\sigma ^{2}, \phi )$. The annualized linear combination of the sine and cosine functions and quarterly a year were used to model the malaria seasonality in addition to covariates for which higher incidence was observed mainly from September to December, following the main rainy seasons, and from March to May, after minimal rainy seasons [25]. These seasonality components were incorporated following observed malaria trends and some literature on the malaria pattern in the country [12, 26]. Then, using the following formula, the forecasts can be generated for each $i^{th}$ district at time t.

$$\begin{aligned} \lambda _{i}(t)=\int _{t}\hat{\lambda _{it}} \end{aligned}$$

where $i=1,2,\dots , 149; t = 1, 2,...,70$, each integer identifies a month, from August 2013 to May 2019. Also, $\hat{\lambda }_{it}$ is the mean of the predictive distribution of intensity at time t for each district. After that, the integrals can be approximated using the MCMC method and then forecast the incidence for the next 12 months using predicted incidence.

### Cross-validation

First, all of the data sets have been divided into training and test sets to evaluate the model’s effectiveness in predicting future outcomes. After that, the model’s performance was evaluated in terms of how well they were able to forecast incidence and estimate the accompanying uncertainty for the 12 months. Finally, by holding out the case reports of 12 months from June 2018 to May 2019 that are accessible, the model is fitted to the remaining data set i.e. from August 2013 to May 2018. Using the root-mean-square error, mean absolute error, and coverage probability, models’ ability to predict outcomes for each of the 12 months are presented.

$$\begin{aligned} RMSE_{t}= & {} \sqrt{\frac{1}{149}{\sum _{i=1}^{149} \left( \lambda ^{emp}_{it}- \hat{\lambda }_{it}\right) ^{2}}}\\ MAE_{t}= & {} \frac{1}{149}{\sum _{i=1}^{149} \left| \lambda ^{emp}_{it}- \hat{\lambda }_{it}\right| }\\ CP_{t}= & {} \frac{1}{149}\sum _{i=1}^{149}I \left( \hat{\lambda }_{it}^{0.025}<\lambda ^{emp}_{it}<\hat{\lambda }_{it}^{0.975} \right) \,, \end{aligned}$$

where $\lambda ^{emp}_{it}$ is the true observed incidence of $i^{th}$ district in the test set at time $t=1,\dots , 12$; $\hat{\lambda }_{it}$ is the predicted mean incidence; and $I \left( \hat{\lambda }_{it}^{0.025}<\lambda ^{emp}_{it}<\hat{\lambda }_{it}^{0.975} \right)$ is an indicator function that takes value 1 if $\hat{\lambda }_{it}^{0.025}<\lambda ^{emp}_{it}<\hat{\lambda }_{it}^{0.975}$ and 0 otherwise, with $\hat{\lambda }_{it}^{0.025}$ and $\hat{\lambda }_{it}^{0.975}$ corresponding to the quantiles; 0.025 and 0.975 of the predictive or posterior distribution for $\lambda _{it}$, respectively.

Finally, a variogram was also used to validate the compatibility of the fitted correlation function to the data by simulating 60,000 data sets under the fitted model. Variogram-based graphical validation was used to check fitted temporal correlation in each district using a PrevMap R package [27]. Furthermore, RMSE is used to evaluate the prediction performance of models with and without covariates. One can further see the variogram-based validation in [24, 27]. PrevMap package in R [27] was used to analyze the data.

## Results

**Fig. 2:**
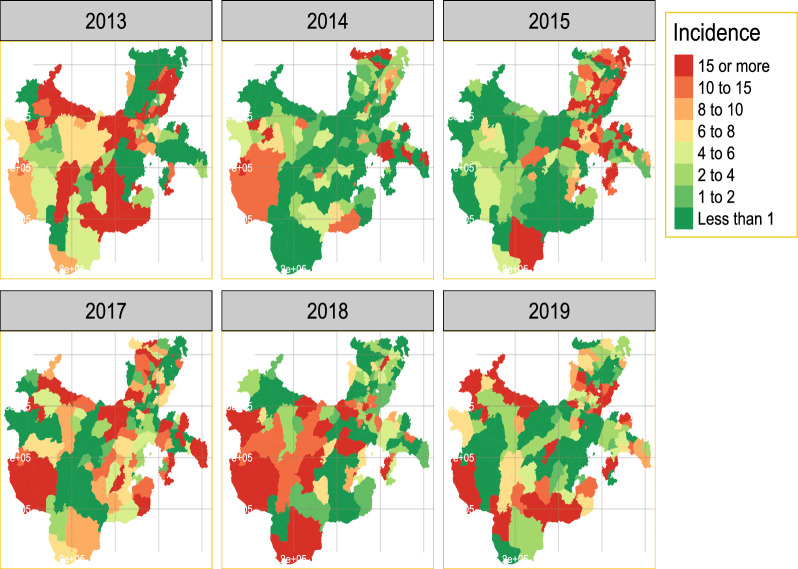
Distribution of yearly aggregated observed incidence of *P. falciparum* in all districts of Southern Ethiopia from August 2013 to May 2019 per 1000 population of all age groups

As shown in Fig. 2, the incidence of malaria changes with time and space, with an increased incidence observed in the western parts of the region. Additionally, the occurrence varies over time, with 2013, 2017, and 2018 respectively seeing the highest numbers of cases. Space-time modelling is useful for comprehending such variability and forecasting future trends.

The distribution of the incidence varies over the districts with higher incidences observed in districts found in the western region and moderate incidences found in the districts found in central areas of the region as was shown in Fig. 2.

**Fig. 3:**
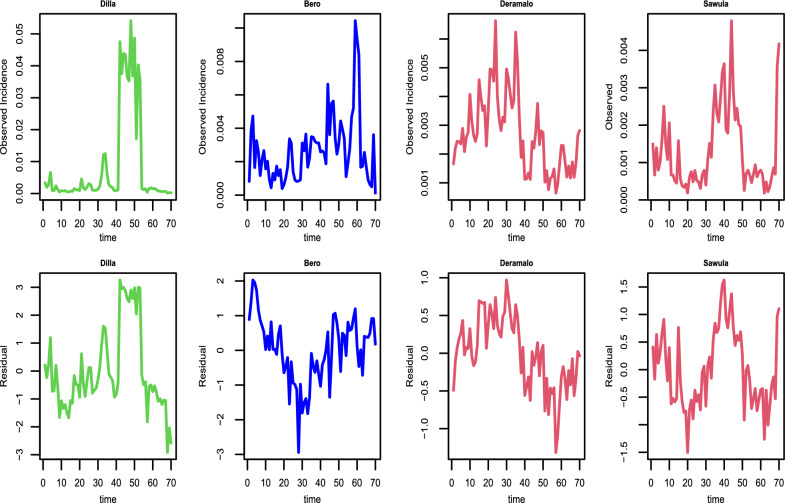
Distribution of observed incidence [upper panel] and residuals from generalized linear mixed model [lower panel] for selected districts from August 2013 to May 2019 by *P. falciparum* in Southern Ethiopia

For some of the randomly chosen districts, the plot of the observed incidences and residuals over time as depicted in Fig. 3 was provided. The illustration shows that the incidence distribution pattern alters with time and space. In particular districts like Bero and Daramalo, there is a signal of an increase in incidence as demonstrated in Fig. 3. On the other hand, the distribution of residuals further revealed that incidences vary across districts. As a result, space-time modelling is important to get further insight into the distribution of the incidences in each district of the region.

**Fig. 4:**
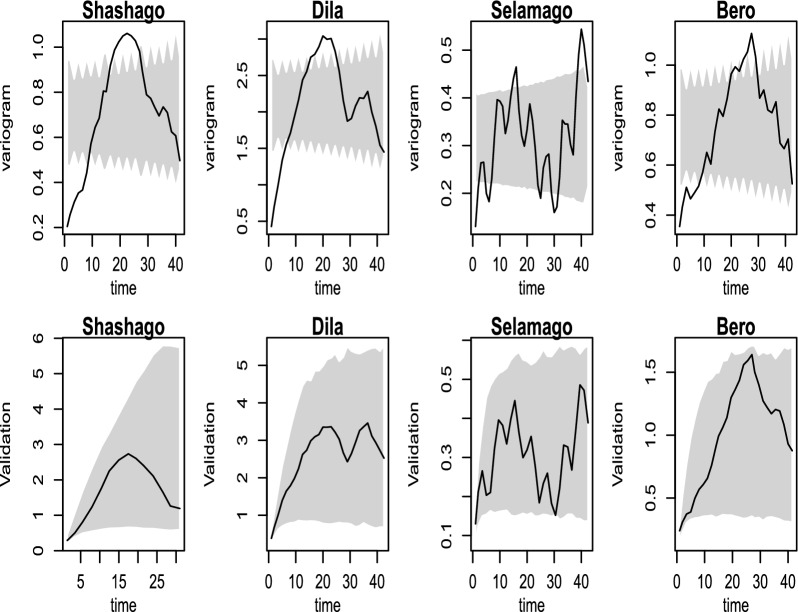
The figures show the outcomes of the Monte Carlo methods applied to test the temporal independence hypothesis (upper panels) and the data compatibility hypothesis (bottom panels) for each district. The 95% confidence region is represented by the shaded areas for each hypothesis. The solid lines represent the empirical variogram for each time bin. Using *P. falciparum* count data from Southern Ethiopia, the theoretical variograms derived using the least squares (solid lines) and maximum likelihood (dashed lines) approaches are displayed in the lower panel

According to the outcome in the lower panel of Fig. 3, it is wise to model the residuals in each administrative district over time since they fluctuate in distribution. There is a signal of increment of the incidences in districts like Deguna-Fanigo, Bero, and Sawula despite the residuals varying over time. The residuals from the non-spatial regression model show the existence of temporal correlation as it was shown in the upper panel of Fig. 4. Also, the incidence is temporally correlated, as it was depicted in the variogram at various time bins in the upper panel of Fig. 4. This is because the observed incidences are out of the confidence bound as shown in the upper panel of Fig. 4 indicating the existence of temporal correlation which decreases as increases in time. Thus, developing a district-level malaria epidemic early warning system is a smart place to start identifying variability as well as epidemics throughout space and time.

Notable malaria cases by genus *P. falciparum* were observed in 2013, 2015, and 2018 across the districts. On the other hand, several districts like Boricha, Loko Abaya, Wenago, Humbo, Konta, Hamer, Basketo, Yemi, and Surima showed peak cases in the year 2013. On the other hand, only some of the districts in Sidama region, Hadiya and Gambata tamboura Zone exhibited any significant malaria incidence. An increase in malaria cases was also observed from 2018 to 2019 in several districts of the region. Therefore it should be very important to model the incidence to anticipate the variation over space and time.

## Model comparison and validation

To validate our models, (1) a variogram-based approach to determine whether the fitted correlation function was compatible with the data was incorporated. Several variograms from the fitted model had been simulated, and then compared to the estimated empirical variogram derived from the data. The estimated empirical variogram, as shown in the lower panel of Fig. 4, completely falls within the 95% confidence interval of the simulated empirical variograms, confirming that the adopted correlation function is appropriate with our data.

(2), then, out-of-sample validation by taking 12-month data as a test set as shown in Table 1 was considered. The result indicates that the method produces a small prediction error with a coverage probability around 75% on average which is not much further apart from 95% confidence interval. Thus, the fitted model is important in predicting disease risk with minimum prediction error and coverage probability closer to 95%.

**Table 1:** Summary of out-of-sample accuracy: root mean square error (RMSE): Mean absolute error (MAR) and coverage probability (CV) obtained for the 12-month validation set data averaged to all districts

Valid	1	2	3	4	5	6	7	8	9	10	11	12
RMSE	0.046	0.022	0.058	0.023	0.013	0.017	0.018	0.039	0.019	0.015	0.015	0.016
MAE	0.012	0.009	0.012	0.009	0.007	0.008	0.009	0.011	0.008	0.008	0.009	0.009
CVP	78.174	75.919	69.154	65.396	74.416	75.919	66.899	72.161	70.658	71.409	69.154	67.651

Finally, the researcher evaluates the importance of including covariates in predicting space-time variation of disease risk. This was done by comparing the prediction performance of models with and without covariates. The result indicates that including covariates is very important in predicting disease as it was shown in Table 2 with smaller RMSE. Adding climatic variables to the model significantly improved the model fit in the majority of the districts with smaller RMSE, see Table 2.

**Table 2:** Comparison of models using: root mean square error (RMSE): obtained with covariates (1) and without covariates (2) for some selected Districts

Districts	RMSE1	RMSE2	Districts	RMSE1	RMSE2
Wolkite	0.036	0.06	Sheshage	0.0053	0.0054
Dila	0.0092	0.0095	Deguna-Fanigo	0.01	0.012
Selamago	0.025	0.03	Dasenech	0.012	0.02
Arbaminch	0.018	0.038	Bero	0.05	0.06
Hawassa	0.002	0.003	Benatsemay	0.035	0.041

**Table 3:** Parameter estimates of the models and their 95% confidence interval for some selected Districts in the Region

Districts	Parameter	Estimate	Districts	Estimate
Wolkite	$\beta _{0}$	−6.95 (−9.26, −4.64)	Sheshage	−7.13 (−11.12, −3.14)
	$$\beta _{1}sin(2*pi*t/12)$$	0.101 (0.065, 0.138)		0.142 (0.109, 0.174)
	$$\beta _{2}cos(2*pi*t/12)$$	0.417 (0.367, 0.467)		−0.224 (−0.264, −0.183)
	$$\beta _{3}sin(2*pi*t/4)$$	−0.178 (−0.360, 0.003)		−0.163 (−0.331, 0.006)
	$$\beta _{4}cos(2*pi*t/4)$$	0.134 (0.099, 0.169)		0.085 (−0.058, 0.228)
	$\beta _{5}log.precipitation$	0.223 (0.105, 0.341)		0.307 (0.207, 0.407)8
	$\beta _{6}temperature$	0.222 (0.163, 0.282)		0.368 (0.247, 0.488)
	$\beta _{7}humidity$	0.123 (0.093, 0.154)		0.186 (0.161, 0.211)
	$\beta _{8}NTL$	0.011 (0.010, 0.012)		−4.860 (−6.525, −3.195)
	$\sigma ^{2}$	0.933 (0.012, 1.853)		0.890 (0.369, 1.412)
	$\phi$	5.806 (5.640, 5.972)		6.976 (6.832, 7.120)
Dilla	$\beta _{0}$	−6.31 (−14.03, 1.42)	Selamago	−4.425 (−16.326, 7.476)
	$$\beta _{1} sin(2*pi*t/12)$$	−0.097 (−0.679, 0.484)		0.089 (−0.143, 0.321)
	$$\beta _{2}cos(2*pi*t/12)$$	0.410 (0.339, 0.482)		0.141 (−0.180, 0.463)
	$$\beta _{3} sin(2*pi*t/4)$$	−0.039 (−0.065, −0.012)		0.104 (0.072, 0.137)
	$$\beta _{4}cos(2*pi*t/4)$$	0.071 (0.043, 0.099)		0.278 (0.130, 0.426)
	$\beta _{5}log.precipitation$	0.292 (0.170, 0.713)		0.776 (0.266, 2.015)
	$\beta _{6}temperature$	0.750 (0.488, 1.011)		0.132 (0.112, 0.152)
	$\beta _{7}humidity$	0.133 (0.091, 0.175)		0.009 (-0.009, 0.027)
	$\beta _{8}NTL$	0.000 (−0.001, 0.002)		3.495 (−1.148, 8.138)
	$\sigma ^{2}$	2.326 (1.967, 2.684)		0.223 (0.060, 0.385)
	$\phi$	5.939 (5.783, 6.095)		1.524 (1.056, 1.991)
Bero	$\beta _{0}$	−9.494 (−15.047, −3.941)	Dasenech	−7.890 (−13.250, −2.531)
	$$\beta _{1} sin(2*pi*t/12)$$	−0.154 (−0.486, 0.177)		0.422 (0.374, 0.470)
	$$\beta _{2}cos(2*pi*t/12)$$	0.330 (0.030, 0.630)		0.125 (0.062, 0.188)
	$$\beta _{3} sin(2*pi*t/4)$$	−0.066 (−0.20, 0.069)		−0.014 (−0.227, 0.198)
	$$\beta _{4}cos(2*pi*t/4)$$	0.011 (−0.134, 0.156)		−0.119 (−0.364, 0.126)
	$\beta _{5}log.precipitation$	0.634 (0.245, 1.022)		2.099 (0.507, 3.691)
	$\beta _{6}temperature$	0.083 (0.012, 0.155)		0.192 (0.015, 0.370)
	$\beta _{7}humidity$	−0.005 (−0.021, 0.010)		0.023 (−0.006, 0.052)
	$\beta _{8}NTL$	−1.200 (−2.395, −0.004)		−0.625 (−8.857, 7.607)
	$\sigma ^{2}$	0.768 (0.475, 1.061)		1.024 (0.414, 1.633)
	$\phi$	5.393 (5.239, 5.548)		2.863 (2.592, 3.134)
Arbaminch	$\beta _{0}$	−4.755 (−7.924, −1.585)	Hawassa	−6.455 (−8.646, −4.264)
	$$\beta _{1} sin(2*pi*t/12)$$	−0.329 (−0.577, −0.080)		0.334 (0.132, 0.535)
	$$\beta _{2}cos(2*pi*t/12)$$	−0.070 (−0.097, −0.044)		0.085 (−0.156, 0.327)
	$$\beta _{3} sin(2*pi*t/4)$$	0.106 (−0.002, 0.215)		0.020 (−0.082, 0.123)
	$$\beta _{4}cos(2*pi*t/4)$$	0.118 (0.014, 0.223)		0.084 (−0.005, 0.172)
	$\beta _{5}log.precipitation$	0.411 (0.256, 0.567)		0.605 (0.197, 1.012)
	$\beta _{6}temperature$	0.389 (0.290, 0.487)		0.012 (-−0.040, 0.063)
	$\beta _{7}humidity$	−0.037 (−0.055, −0.018)		−0.008 (−0.024, 0.007)
	$\beta _{8}NTL$	0.021 (0.020, 0.021)		0.000 (0.000, 0.001)
	$\sigma ^{2}$	0.538 (0.140, 0.936)		0.784 (−1.121, 2.689)
	$\phi$	7.497 (7.375, 7.619)		17.129 (17.037, 17.220)

The model parameters’ maximum likelihood estimates are shown in Table 3 which indicates that the incidence varies throughout the year in some of the districts and seasonally in other districts. Precipitation, temperature, humidity, and nighttime light are significantly associated with the incidence in the majority of the district. For instance, an increase in precipitation is significantly associated with 0.223 (0.105, 0.341), 0.307 (0.207, 0.407), 0.292 (0.170, 0.713), 0.776 (0.266, 2.015), 2.099 (0.507, 3.691), 0.411 (0.256, 0.567), 0.634 (0.245, 1.022), 0.605 (0.197, 1.012) increase in malaria risk in Weleikite, Shashago, Dila, Selamago, Dasenech, Arbaminch, Bero and Hawassa districts respectively. Whereas, an increase in temperature, humidity, and nighttime light is also significantly associated with malaria risk in some of the districts Table 3.

When combined with the distribution of residual in Fig. 3, variance $sigma^{2}$, which is significant in the majority of the districts, shows the variability of the incidence over time. For the districts shown in Table 3, it is estimated that the practical range of the temporal correlation is $log(20) \times \hat{\phi }$, i.e. $log(20) \times 1.524 \approx 4.6$ months and $log(20) \times 17.13 \approx 51.3$ months respectively in Selamago and Hawassa. The practical range is defined as the months beyond which the temporal correlation is below 0.05. In Selamago and Hawassa, respectively, the 95% confidence interval for the practical range ranges from 3.16 to 6 months and between 50 and 52 months.

**Fig. 5:**
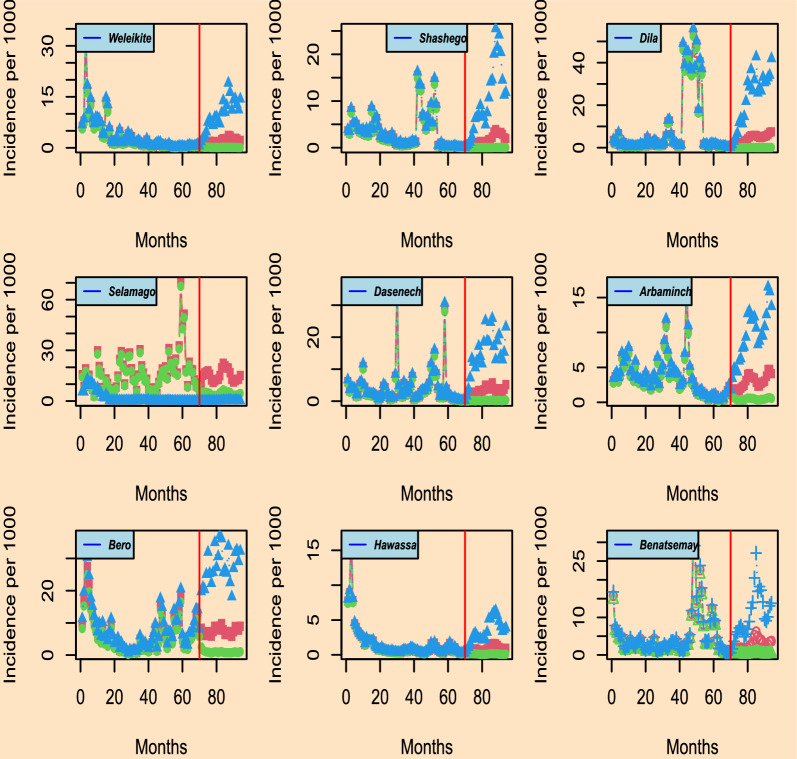
Prediction results for the districts of Weleikite, Shashego, Dila, Selamago, Duguna-fango, Dasenech, Arbaminch, Bero, and Hawassa overall months from 2013 and forecast for the 24 months. The plot shows predictive inference and associated 95% confidence interval for *P. falciparum*

Even though no significant outbreaks were observed in the study area during the study period, there were times when notable incidences were observed from September to November 2013 in the Woleikite, November to December 2016 in Shashego, January to March 2018 in Dila, June to August 2018 in Selamago, October to November 2015 and January to February 2017 in Arbamich and early 2013 in Hawassa city Fig. 5. The right panel of Fig. 5 indicates the forecast for the next 24 months. The forecast indicates, the re-emergence of the incidence in the area as indicated in Fig. 5. In general, the decreasing trend of the incidence was observed till the end of 2018 with an unstable rate, and there is a signal of re-emergency starting from 2019. For the last 24 months of the time series, the forecasted incidences were provided, and the result indicates a signal of the outbreak for most districts in the region.

**Fig. 6:**
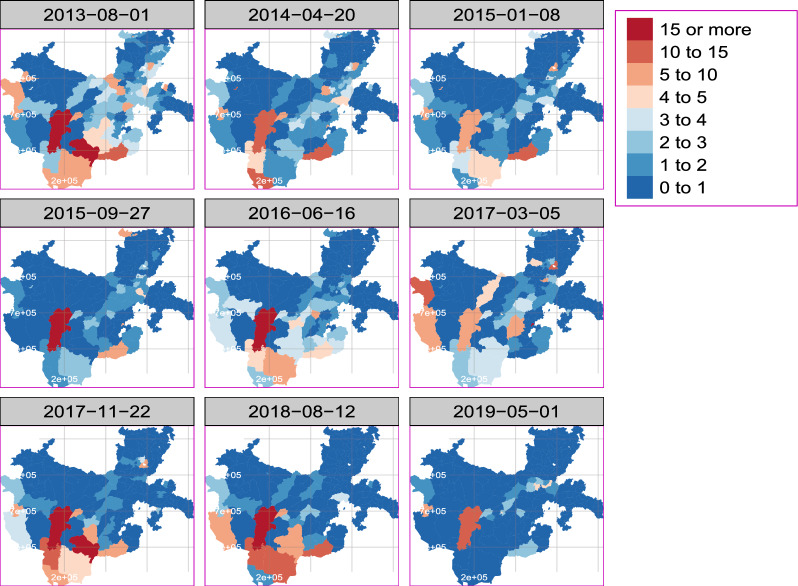
Prediction incidence for the selected months of the years for all districts for *P. falciparum* incidence per 1000 population

The variation in incidences throughout time and space is depicted in the prediction map in Fig. 6. The southwest’s Bero, Selamago, Hamer, and Dasenech districts also saw higher incidences. Moreover, a considerable number of districts in the region’s northwest and centre have a moderate to high incidence of malaria. Districts in the north and southeast, on the other hand, exhibited a lower incidence.

**Fig. 7:**
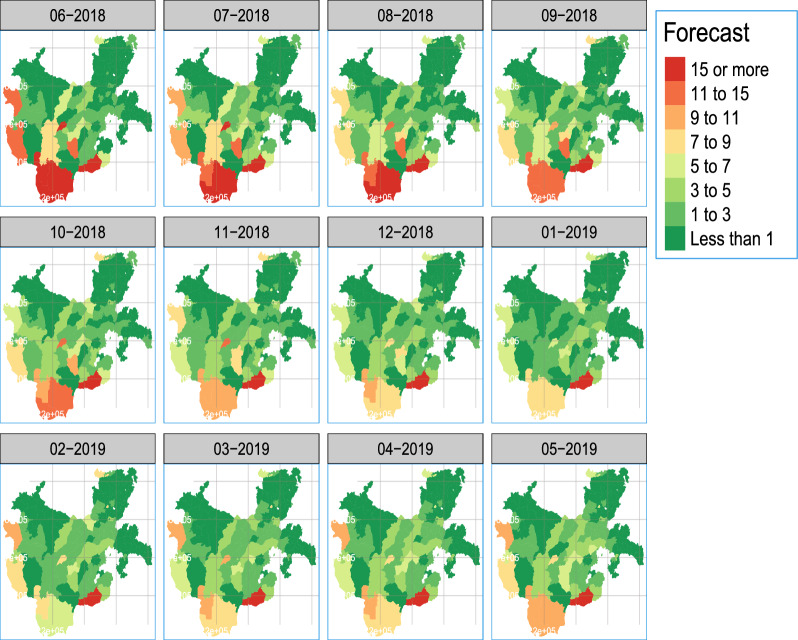
Forecasted map of *P. falciparum* incidence per 1000 population in the region from June 2018 to May 2019 for all districts

## Discussion

According to our study results, it would be ideal to establish a malaria epidemic early warning system in each administrative district to better understand the geographic distribution of cases. As noted by [28] in one of the local administrative districts, the incidence varies among the districts. This highlights how establishing an early warning system for epidemics in each district enables the local administration to act right away to address the issue rather than constantly waiting for a solution from outside sources. Similarly to [1, 12] report, the temporal trend in the region varies over time from district to district as displayed in Fig. 3. The transmission is not consistent; rather, it varies from district to district Figs. 2, 5, and 6, with some exhibiting a similar pattern.

To forecast the pattern and re-emergence of malaria as shown in Fig. 7 across several districts, a time series model-based epidemic early warning system for malaria is a promising choice. Yet, as demonstrated in Table. 2, incorporating variables dramatically improved prediction performance. However, if an outbreak is only briefly observed before going away, the value of EWSs as a forecasting tool for policymakers may be not used. The forecasts as shown in Figs. 7 and 5 indicate cases are emerging in some of the districts in the region. As model primarily forecasts a nonlinear increase as was shown in Fig. 5, which is characteristic of the seasonal pattern of the illness risk and is consistent with [5, 16].

Even though this study sheds light on the resurgence of the incidents in various districts, the applicability of its findings is frequently constrained by the poor quality of the available data [29]. This is due to the effect of reporting bias affecting surveillance data in low-resource environments. When districts are spread out and the importance of the spatial correlation is uncertain, time series modelling might be useful in identifying these situations. The modelling of such cases independently provides superior information regarding the incidences for specific districts, however, as some districts may show distinctive tendencies when compared to other districts.

Increases in rainfall have a considerable impact on malaria risk, as shown in Table 3, which is consistent with earlier research [16, 30]. This may be because mosquitoes breed near water bodies, where they are occasionally visible after a very strong rainstorm. This is typical in the majority of the districts in the area, as it has been widely discussed in various works of literature [31]. On the other hand, there is a strong correlation between the risk of malaria and the temperature. Malaria risk rises by 0.22 in Woleikte, 0.37 in Shashego, 0.750 in Dila, 0.132 in Selamago, 0.083 in Bero, 0.19 in Dasenech, 0.39 in Arbaminch, and 0.012 in Hawassa city with each degree of temperature increase. This implies that a rise in temperature and rainfall correlates with [28] and has a strong favourable impact on all districts with moderate to high malaria cases. In Woleikte, Shashego, and Dila districts, respectively, an increase in humidity is similarly linked to increases in malaria risk of 0.123, 0.186, and 0.133%; however, this association is not statistically significant in some of the other districts. On the other hand, a rise in nighttime light is adversely correlated with 0.86 in Shashsgo, 0.2 in Bero, and 0.625 in Dasenech increase in malaria risk. Yet there were also discovered positive connections in other districts as presented in Table 3.

As a signal of re-emergency is noticed in some districts in the region, malaria risk prediction with greater accuracy is currently crucial in nations like Ethiopia as seen in Fig. 5. Someone might take into account a different option that aids in the detection of hotspots while designing such a system. Because infectious diseases like malaria fluctuate with both space and time with notable influence of nearby areas, it may therefore be very important to build a system that considers spatial correlation into account.

Fitting more precise malaria models for the future, not only to revisit our findings but also to precisely address various questions about the underlying trend in districts and various malaria cases with multiple *Plasmodium* species could be important. A promising path has recently been opened up by the development of discrete and continuous spatial models for infectious disease dynamics [14]. Future research anticipates going into more detail on some of the important drivers and aspects that this study did not sufficiently explore regarding the prevalence of malaria in Southern Ethiopia. The results obtained highlight the value of dynamically identifying districts with elevated risks, but additional modelling is needed to fully understand the spatio-temporal variations of malaria risk in the region.

## Conclusion

Space-time modelling of malaria risk is crucial for describing the aetiology of the disease and directing decision-making at the lower administrative levels. A re-emergency signal was seen a few months/years ago with an unstable rate despite the incidence having been on the decline for the past few years. Districts found in the southwest have detected higher incidence that varies with time. When incidence heterogeneity increases, it is important to address “bottlenecks” such as dealing with persistent foci, subsequent re-emergencies, and parasite development areas. In a changing climate, sustainable and adaptive plans should now be guided from an informed local level.

## Data Availability

The data presented in this study are available on request from the corresponding author. The data are not publicly available due to the data-sharing policy of the Ethiopian Public Health Institute. Temperature and precipitation data sets were extracted freely from (worldclim.org/data/monthlywth.html and worldclim21.html), Relative Humidity was derived from ERA-Interim global atmospheric reanalysis and Nighttime light (NTL) obtained from NOAAs National Centers for Environmental Information,(https://ngdc.noaa.gov/eog/ viirs/index.html).
